# Unveiling the Effect of Low pH on the SARS-CoV-2 Main Protease by Molecular Dynamics Simulations

**DOI:** 10.3390/polym13213823

**Published:** 2021-11-05

**Authors:** Haruna Luz Barazorda-Ccahuana, Miroslava Nedyalkova, Francesc Mas, Sergio Madurga

**Affiliations:** 1 Materials Science and Physical Chemistry Department & Research Institute of Theoretical and Computational Chemistry (IQTCUB), University of Barcelona, 08028 Barcelona, Spain; hbarazba20@alumnes.ub.edu; 2 Vicerrectorado de Investigación, Universidad Católica de Santa María, Arequipa 04000, Peru; 3 Department of Inorganic Chemistry, University of Sofia “St. Kl. Okhridski”, 1164 Sofia, Bulgaria; mici345@yahoo.com

**Keywords:** SARS-CoV-2 Mpro, pH, molecular dynamics, docking analysis, PF-00835231

## Abstract

(1) Background: Main Protease (Mpro) is an attractive therapeutic target that acts in the replication and transcription of the SARS-CoV-2 coronavirus. Mpro is rich in residues exposed to protonation/deprotonation changes which could affect its enzymatic function. This work aimed to explore the effect of the protonation/deprotonation states of Mpro at different pHs using computational techniques. (2) Methods: The different distribution charges were obtained in all the evaluated pHs by the Semi-Grand Canonical Monte Carlo (SGCMC) method. A set of Molecular Dynamics (MD) simulations was performed to consider the different protonation/deprotonation during 250 ns, verifying the structural stability of Mpro at different pHs. (3) Results: The present findings demonstrate that active site residues and residues that allow Mpro dimerisation was not affected by pH changes. However, Mpro substrate-binding residues were altered at low pHs, allowing the increased pocket volume. Additionally, the results of the solvent distribution around Sγ, Hγ, Nδ1 and Hδ1 atoms of the catalytic residues Cys145 and His41 showed a low and high-water affinity at acidic pH, respectively. It which could be crucial in the catalytic mechanism of SARS-CoV-2 Mpro at low pHs. Moreover, we analysed the docking interactions of PF-00835231 from Pfizer in the preclinical phase, which shows excellent affinity with the Mpro at different pHs. (4) Conclusion: Overall, these findings indicate that SARS-CoV-2 Mpro is highly stable at acidic pH conditions, and this inhibitor could have a desirable function at this condition.

## 1. Introduction

The COVID-19 pandemic is still an ongoing major health threat for the whole world, caused by severe acute respiratory syndrome coronavirus 2 (SARS-CoV-2) [1,2,3]. The coronavirus family enters the host by fusion with the plasma or endosomal membranes [4,5]. This mechanism has been used by many positive-strand RNA viruses like the influenza A virus (IAV) [6,7], the human immunodeficiency virus (HIV) [8,9], coronaviruses and others. Among the therapeutical targets of these types of viruses are proteases, classified according to their catalytic site as cysteine proteases [10], serine proteases [11] aspartic proteases [12,13] and metalloproteases.

In the SARS-CoV-2 entry mechanism, the coronavirus spike protein binds to ACE2 receptors found on the surface of many human cells. The virus enters the cytoplasm by endocytosis, then its genetic material, a single-stranded RNA, is released into the cytoplasm. This material encoded two polyproteins (pp1a and pp1ab) which are necessary for viral replication and transcription [14,15,16]. The proteolytic process was carried out by the SARS-CoV-2 Main Protease (Mpro), a cysteine protease with a catalytic dyad that compromises two principal residues, Cys145 and His41 [17,18,19,20,21,22]. This protein is essential in the life cycle of the virus and is considered a relevant target against the replication of the coronavirus. The catalytic mechanism among coronavirus cysteine proteases involves either using a catalytic dyad to perform a nucleophilic attack that covalently links the protease to the substrate protein, releasing the first half of the product. This covalent acyl-enzyme intermediate is then deacylated by an active water molecule releasing the second half of the product [23,24].

**Figure 1:**
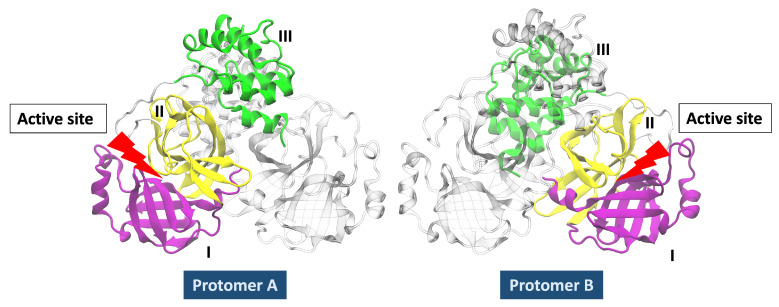
SARS-CoV-2 Mpro, PDB ID: 5RE4. Domain I in purple colour, domain II in yellow colour and domain III in green colour.

SARS-CoV-2 Mpro is active in its dimeric conformation, but the SARS-CoV-1 Mpro is found as a mixture of monomer and dimers in solution [25]. SARS-CoV-1 Mpro has been crystallised in different pH-dependent conformations suggesting flexibility, particularly around the active site [26]. The protease is highly conserved in all coronaviruses, including SARS-CoV-2, so targeting either dimerisation or enzymatic activity may give rise to drugs that can target multiple coronaviruses, known and yet unknown [27,28]. In Figure 1, the three-dimensional structure representation of SARS-CoV-2 Mpro (approx. 33kDa of total weight) shows two identical protomers (homodimer conformation). Each protomer has three domains, domain I (residue 10 to 99), domain II (residue 100 to 182), and domain III (residue 198 to 303); a cluster of five α-helices characterises this last domain. Domain II is connected to domain III via a longer loop.

Many macromolecular properties in the coronavirus family are pH-dependent [4,29]. As a starting point, it is important to mention that there are studies on the effect of pH in SARS-CoV-1 Mpro, showing the high proteolytic activity of SARS-CoV-1 Mpro around the physiological pH (7.0–7.6) [26,30,31,32,33]. The probability of the pH-activity profile of the SARS-CoV-1 Mpro was determined by protonation of His163 (inactivation at acidic pH) and deprotonation of His172 [26]. This behaviour is different for conformational stability; some viral proteases have shown their biological role at low pHs, as shown in the endogenous viral proteases from porcine coronavirus are active in a low-pH environment [34].

The SARS-CoV-2 Mpro activity is thought to exhibit a bell-shaped pH-rate profile as determined for the SARS-CoV-1 Mpro. For SARS-CoV-1, fitting kinetic data to model equations provides rate constants and two pKa values (6.2–6.4, 7.7–8.3) that presumably correspond to the catalytically competent His41 and Cys145 side chains [35,36]. Further understanding of pH effects can be gained from theoretical pKa estimations for all the titratable amino acids of Mpro, which have been also employed to determine their most likely protonation states [31]. Another recent work has demonstrated that the structure of SARS-CoV-2 Mpro shows pH dependence. At basic pH, the Mpro structure was less stable, and as pH becomes more acidic, the protein gets destabilised. Moreover, the SARS-CoV-2 Mpro showed maximum stability at neutral pH but in the other pHs analysed other than neutral pH, dimerisation and S1 binding pocket were found to be affected [37].

Moreover, due to the high homology between SARS-CoV-1 Mpro and SARS-CoV-2 Mpro, similar behaviour is expected. Indeed, the coronavirus family is characterised as an enveloped virus that may or may not be low pH dependent [26,38,39]. It has been reported that higher levels of ACE2 in tissues could be the consequence of some of the comorbidities associated with severe SARS-CoV-2 infection [40]. They showed that the acid pH associated with Barrett’s oesophagus patients might drive an increased expression of ACE2. In addition, they studied human monocytes cultured in acid pH, observing an increased ACE2 expression and higher viral load upon SARS-CoV-2 infection [40]. A recent review shows that acidosis is generally associated with the severity of SARS-CoV-2. Lactic acidosis produces an excess of the levels of lactate, which is associated with a reduction of pH [41]. The associated enzyme, lactate dehydrogenase (LDH), which transforms pyruvate to lactate, could be selected as one biomarker, as high LDH levels have been associated with worse outcomes in patients with the viral infection [42]. The excess of lactate, a stronger acid than pyruvate, leads to a decrease in the pH of the medium. Herein, we investigate the effect of acidic conditions on the pH-dependent conformational properties of SARS-CoV-2 Mpro, which may be involved in inhibition processes associated with severe SARS-CoV-2 infections. Currently, the literature shows us several studies of the effect of pH by docking calculations. These studies have shown that the pH dependence of the enzyme activity may reflect structural changes in the catalytic dyad [43,44,45,46,47,48,49]. For instance, Drazic et al. tested the influence of different pH values on substrate cleavage velocity and found no significant impact in the pH range from 7.0 to 8.0 [50]. Several publications described the influence of pH on the enzyme activity of the SARS-CoV-1 Mpro, which reported a peak of substrate cleavage at pH 7.0 [26,51], whereas other studies reported the highest processing at around pH 7.5 [31], or pH 8.0 [52,53].

The first analysis by molecular dynamic simulation at pH 7 of SARS-CoV-2 Mpro demonstrated the active site’s flexibility and effects induced by substrate binding [35]. On the other hand, SARS-CoV-2 Mpro showed high thermodynamic stability in a wide pH range and low stability in the presence of salts [45]. With respect to the catalytic site, the most likely hydrolysis mechanism assisted by the coronavirus Mpro, the reactive events would involve the proton transfer from Cys145(SγH) to His41(Nϵ) and the nucleophilic attack of the Sγ atom to the carbonyl group of a Gln. Thus, the general mechanism considers that Cys145 should be protonated and His41 acts as a base, as in the case of the neutral pH [35].

Verma et al. analysed the proton-coupled dynamics of SARS-CoV-2 Mpro that represented the first step toward the mechanistic understanding of the proton-coupled structure dynamics function at pH 5 to 9 [54]. Likewise, new advances in pH-dependence have shown the importance of appropriate histidine protonation states of SARS-CoV-2 Mpro in the coupling with inhibitors [55]. In the last two years, different SARS-CoV-2 inhibitors have been studied, such as synthetic and natural compounds [28,56,57,58,59,60,61,62,63,64,65,66,67,68,69,70,71].

Our study provides evidence about the relation between flexibility and pH-dependent Mpro, which was observed at the low pH conditions. We applied Semi-Grand Canonical Monte Carlo (SGCMC) method [72,73,74,75] to assign different protein charge distributions, considering the protonation/deprotonation states for each pH. The homemade developed code within our group makes it possible to define different protonation/deprotonation microstates for a particular pH as shown in the work of Barazorda-Ccahuana et al. [76]. One step further was made by analysing the observed conformational changes due to protonation/deprotonation states by atomistic molecular dynamics simulations.

Along with the explored protonation/deprotonation impact presented in an atomistic level for the Mpro in the frame of molecular dynamics simulations, also we stressed the effect of interaction with PF-00835231 as a potent inhibitor of SARS-CoV-2 Mpro by docking calculations at different pHs. The finding of the therapeutic effect of the PF-00835231 has demonstrated promising data or outcomes in Pfizer preclinical studies [19,77,78,79,80,81,82]. Indeed, the SARS-CoV-2 Mpro and PF-00835231 coupling analysis could serve as a model to study other molecules of natural origin targeting SARS-CoV-2.

## 2. Computational Methods

### 2.1. Protonation/Deprotonation States by SGCMC

We have used the SARS-COV-2 Mpro crystal structure reported in the Protein Data Bank (https://www.rcsb.org, accessed on 10 June 2020) by the access code PDB ID: 5RE4. It was determined by the X-ray diffraction method with a resolution of 1.88 Å. The first step was to determine the pKa values of each ionisable residue with the PROPKA v. 3 program [83,84]. Indeed, the summary of pKa values were used for the calculation of protonation states of Asp, Glu, Arg, Lys and His residues, and the C-terminal and N-terminal ends.

In this work, we use the homemade code (see Data Availability Statement) that calculates the protonation and deprotonation states by Semi-Grand Canonical Monte Carlo (SGCMC) procedure based on the free energy associated with the pKa of each *i* titratable residue, this is calculated using the Equation (1) or Equation (2) [72,73,74,75]

(1) $$\Delta G=+k_{B}T\left(ln\left(10\right)\left(pH-pKa_{i}\right)\right)\;\mathrm{Protonation}$$

(2) $$\Delta G=-k_{B}T\left(ln\left(10\right)\left(pH-pKa_{i}\right)\right)\;\mathrm{Deprotonation}$$

In this work, we applied 200,000 steps for protonation/deprotonation of SARS-CoV-2 Mpro residues generating ten independent microstates for five pHs (3 to 7).

### 2.2. Molecular Dynamics Simulation Details

Molecular Dynamics (MD) simulations have been performed for four microstates for each condition of pHs with the Gromacs (Groningen Machine for Chemical Simulations) v. 2019.1 software [85]. AMBER99SB-ILDN [86] force fields have been used in the simulations of SARS-CoV-2 Mpro. The periodic boundary conditions (PBC) [87] were considered to minimise edge effects in a finite system. For each system, the protein was in the centre of a cubic box with a minimum distance of 1.5 nm to the faces of the simulation box. The box system was solvated with TIP5P [88] water model. As well as, the total charge of each microstate was neutralised with Cl${}^{-}$ or Na${}^{+}$ to attain equilibration. Energy minimisation was carried out using the steepest-descent algorithm with 200,000 steps of simulation. WE performed 10 ns MD simulations of equilibrium in the canonical ensemble NVT with position restraint, and the temperature was regulated with the V-rescale thermostat at 309.65 K. Furthermore, we performed the production of simulation in the isothermal-isobaric ensemble with the Parrinello–Rahman barostat with a reference pressure of 1 bar, V-rescale thermostat with 309.65 K and 250 ns time of the simulation. To further corroborate our findings, the conformational ϕ and ψ angles of residues in Mpro were analysed. This analysis of the quality of the structure was solved with the Ramachandran plot in the Molprobity server [89].

### 2.3. Molecular Docking

Molecular Docking calculations were performed using the AutoDock Vina software [90] integrated into the SAMSON molecular design platform as a SAMSON extension available at http://samson-connect.net, accessed on 10 June 2020. The extension provides additional functionality to prepare receptors and ligands easily, dock ligand libraries, analyse and export docking results. We used SAMSON and Vina extensions to configure calculations, export input files, and run docking calculations.

The number of modes was set to 100 with an energy range = 3 kcal/mol (default value). The energy range (kcal/mol) is a maximum energy difference between the best binding mode and the unfavourable one displayed. The energy (affinity) that differs more than 3 kcal/mol from the best mode are not saved among results. In the configuration file, the parameter called “exhaustiveness” was set to 8. This parameter controls how comprehensive will be the search space.

### 2.4. Simulation Data Analysis

The RMSD (Root-Mean Squared Deviation), RMSF (Root-Mean Squared Fluctuation), RG (Radius of Gyration), HB (Number of Hydrogen Bonds), SASA (Solvent Accessible Surface Area) and RDF (Radial Distribution Function) analysis were calculated by Gromacs tools.

Additionally, the Salt Bridges (SB) were analysed, considering the last frame of MD for each microstate with the “Salt Bridges” utility of the Visual Molecular Dynamic (VMD [91]) program. Finally, the plots were made in Gnuplot v. 5.4 [92] and the represented in the manuscript images were created with VMD.

## 3. Results and Discussion

### 3.1. Protonation/Deprotonation States of SARS-CoV-2 Mpro

The molecular structure of SARS-CoV-2 Mpro is a homodimeric structure with approximately 36% of acid/base residues (Asp, Glu, Arg, Lys, His), and almost 8% corresponds to Cys residue distributed throughout the protein structure. For this reason, the analysis of protonation and deprotonation of titratable residues could affect the total charge of Mpro. In this work, different charge distributions are used to assign the protonation state of Mpro acid/basic residues for each pH. For instance, in the case of acidic residues (Glu and Asp) of each microstate, these can be found in the protonated or deprotonated modes for pH studied; as well as for basic residues (His, Lys, and Arg). In the case of the residues of the catalytic dyad (His41 and Cys145), we considered Cys145 protonated and His41 in the neutral state.

Thus, as conventional MD simulations are used, the atomic charges and the associated protonation states are constant throughout the simulation. The effect of charge fluctuation on a pH is approximated by averaging different simulations with different charge distributions. This approach should be a good approximation of the pH effect, as no overall structural changes of the protein are observed that could significantly change the intrinsic acidity constants.

**Figure 2:**
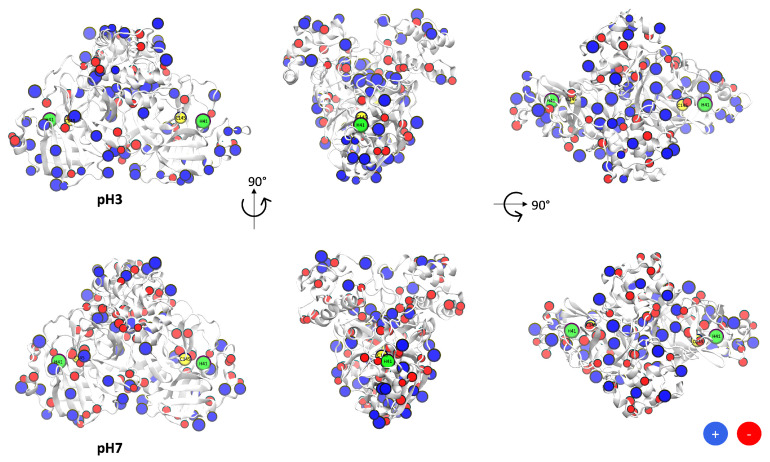
SARS-CoV-2 Main Protease (Mpro) with titratable group in beads representation. Yellow beads (Cys145), green beads (His41), blue beads (basic amino acids) and red beads (acidic amino acids).

For instance, Figure 2 shows the Mpro at pH 3 and pH 7, where the beads of blue colour represent the protonated residues and the red colour the deprotonated residues. We notice that at pH 3 there is a low concentration of red beads, the microstates are more protonated at this condition. In contrast, at pH7 a more equilibrated distribution of red and blue beads are seen in the structure of the Mpro. Whereas, in both states (pH 3 and pH 7) the yellow (Cys145) and green (His41) beads represented the residues from the active site with a neutral charge.

**Figure 3:**
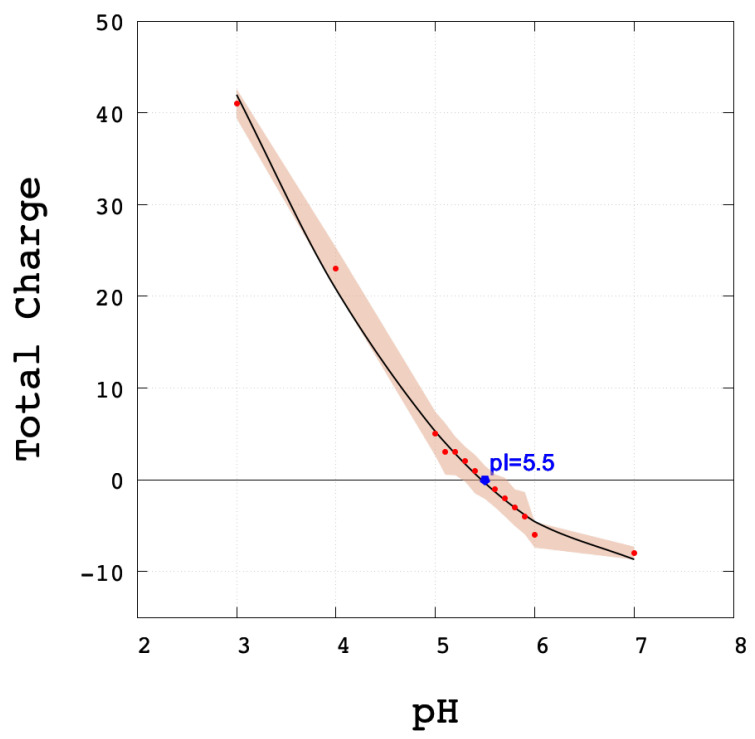
Total charge of Mpro at different pHs. The representation of smooth (colour) indicates the average and the error for each microstate, the estimated computationally pI = 5.5 is in blue colour, and the points were fitted to a polynomial regression line (black line).

Indeed, in Table 1 we evaluate the total charge of 10 microstates generated by the SGCMC method, where the average total charge was +(41 ± 2) (pH 3) and −(8.4 ± 0.7) (pH 7), and zero value around pH 5, as shown in Figure 3. These results allowed us to find the estimated computationally isoelectric point (pI) of SARS-CoV-2 Mpro (pI = 5.5) similar to other studies [93].

Table S1 from Supporting Information shows the distribution of the total charge of the protein among the different titratable residues of the protein. It could be seen that the charge state of acidic residues (Asp and Glu) was the most affected by pH in the studied range, where a large part of these residues at pH 3 was in their protonated state, generating an average of −(9 ± 1) (Asp) and −(4 ± 1) (Glu) partial charge. For pH 4, there are values of −(23 ± 2) (Asp) and −(7 ± 1) (Glu). For the case of pH 5, pH 6 and pH 7, they have had a similar number of Asp and Glu deprotonated. The basic residues (Lys and Arg) were always charged independently of the studied pH. On the contrary, the average total charge of His was +10, +(9 ± 1), +(6 ± 1), +(2 ± 1) and +(1 ± 1) for pH 3, pH 4, pH 5, pH 6 and pH 7, respectively (as shown in Table S1 from Supporting Information). Additionally, we focused on the charge state of two important histidines of the active site, His41 and His172. In general, these two histidines were protonated at pH 3 and pH 4 and deprotonated at pH 5, pH 6 and pH 7 (Table S2 from Supporting Information).

### 3.2. Analysis of Molecular Dynamic Simulation

**Figure 4:**
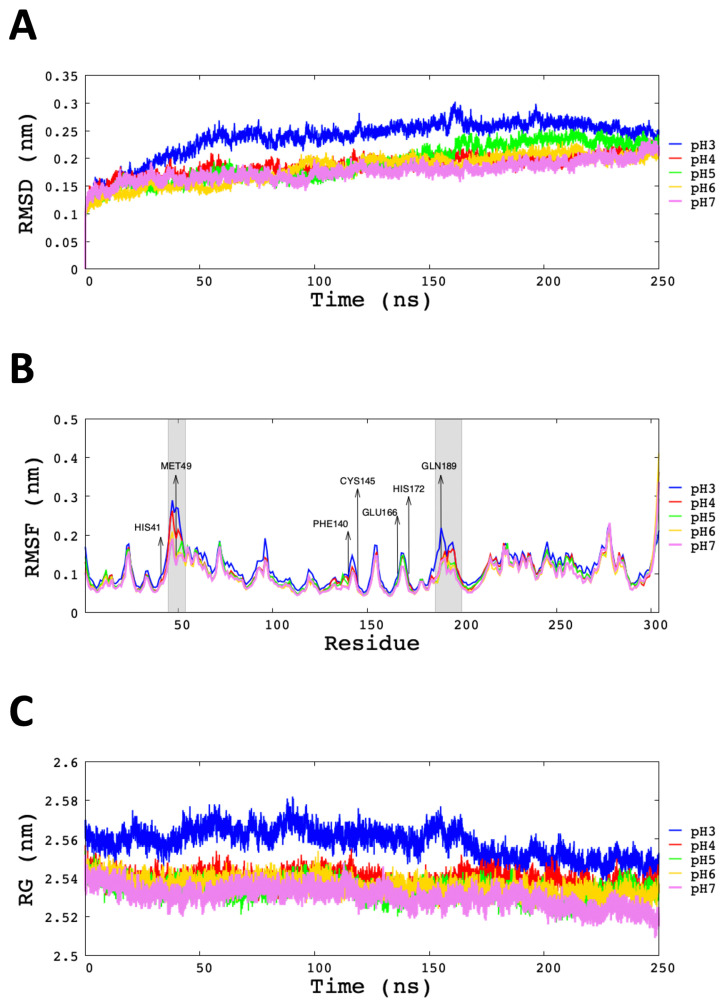
RMSD, RMSF and RG graph of backbone for each pH modelled. (**A**) RMSD presented as a function of time. (**B**) RMSF *per residue* of C-α, highlighting the most important residues of Mpro, during last 100 ns of MD simulations. (**C**) RG analysis of structural compactness.

To envisage the role and the importance of conformational flexibility of the explored cases 20 MD simulations were conducted (4 independent microstates for each of 5 pHs considered). In Figure 4A the RMSD diagram indicates that the Mpro in explicit solvent TIP5P water model starts to be relatively stable around 50 ns at the different conditions of pHs.

It can be seen in Table 2 that the average value of the RMSD at pH 3 was (0.26 ± 0.06) nm; this was the highest value among all simulated pHs. In the range of pHs 4 to 7, the average RMSD value was very similar. In order to analyse the dispersion of RMSD values among different microstates, individual RMSD values are given in Table S3 from Supporting Information.

The RMSF for C-α atoms *per residue* of Mpro were computed at different pHs (Figure 4B). In all cases, the behaviour seems to be similar for all pHs; for example, the catalytic dyad’s residues (His41 and Cys145) presented low fluctuation independently of the studied pH. On the other hand, the average RMSF of the regions that compromise residues 45 to 50 (Thr45, Ser46, Glu47, Asp48, Met49, Leu50) and 186 to 190 (Val186, Asp187, Arg188, Gln189, Thr190) showed us high fluctuation at pH 3 and pH 4 (Figure 4B). Regarding the Glu166 residue, an important residue that participates in the protomers dimerisation, its degree of fluctuation is not affected by the studied pHs. Therefore, the relative stability of global RMSD in conjunction with the relatively low values of RMSF of the main part of the amino acids implies that the global structure of Mpro does not disaggregate at low pH conditions, dimeric conformation is stable.

The RG values determined the compactness of the Mpro and were plotted as a function of time. It can be seen in Table 2 that at pH 3 and pH 4 are obtained the highest RG value 2.55 ± 0.01 and 2.54 ± 0.01, respectively. In contrast, at pH 5, pH 6 and pH 7 the stability and compactness of the protein were similar (approx. 2.53 nm). The SASA analysis of each pH studied is given in Table 2. The average SASA value at pH 3 exhibited increased compared to the other systems ($SASA_{pH3}$ = (268 ± 2) nm${}^{2}$). Therefore, the SASA value increase in average as pH decreases. The temporal evolution of the SASA values at different pHs are shown in Figure S1a from Supporting Information.

However, significant differences could be observed in the number of protein–protein and protein–waters hydrogen bonds. In particular, the average number of hydrogen bonds, protein–protein and protein–waters increase in average as pH increases (Figure S1b,c from Supporting Information show additional information of each pH studied). In summary, these results show that Mpro at pH 3 presented a lower quantity of hydrogen bonds (protein–protein) than other pHs analysed. The fact that Mpro has many aspartic residues led to constraints for hydrogen bonds formation when the residues were protonated at low pH.

**Figure 5:**
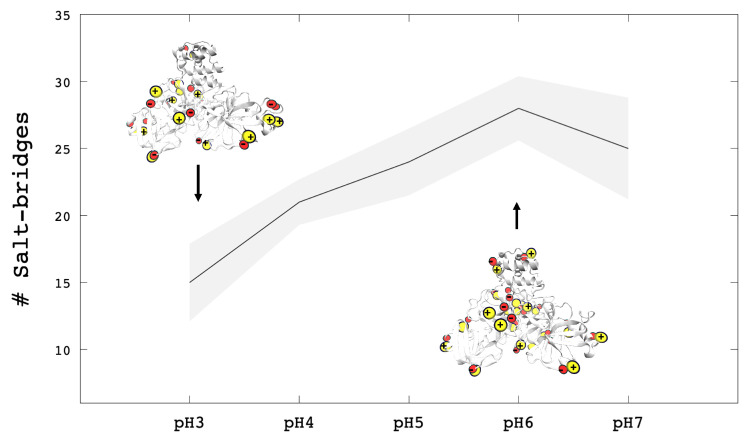
Salt bridges of whole Mpro structure at different conditions of pHs. At low pHs, Mpro is more protonated than pH 6 and pH 7, this graphic show less salt bridges at pH 3. The line-tendency (black) represent the average value and the smooth tendency (gray) represent the statistical error.

Another parameter that determines the stability of Mpro, was the number of salt bridges that depend on the binding of positively and negatively group charges [94]. In this work, the change in the protonated and deprotonated states of Mpro determined that at low pHs exist a loss of salt-bridges. Usually, since the salt bridges depend on the pH, we saw that at pH 3, there is a lower number of salt bridges than the other pHs (Figure 5). Since these systems are further from the isoelectric point (pI = 5.5), the positive and negative charge is unbalanced, so a lower amount of salt bridges is expected.

**Figure 6:**
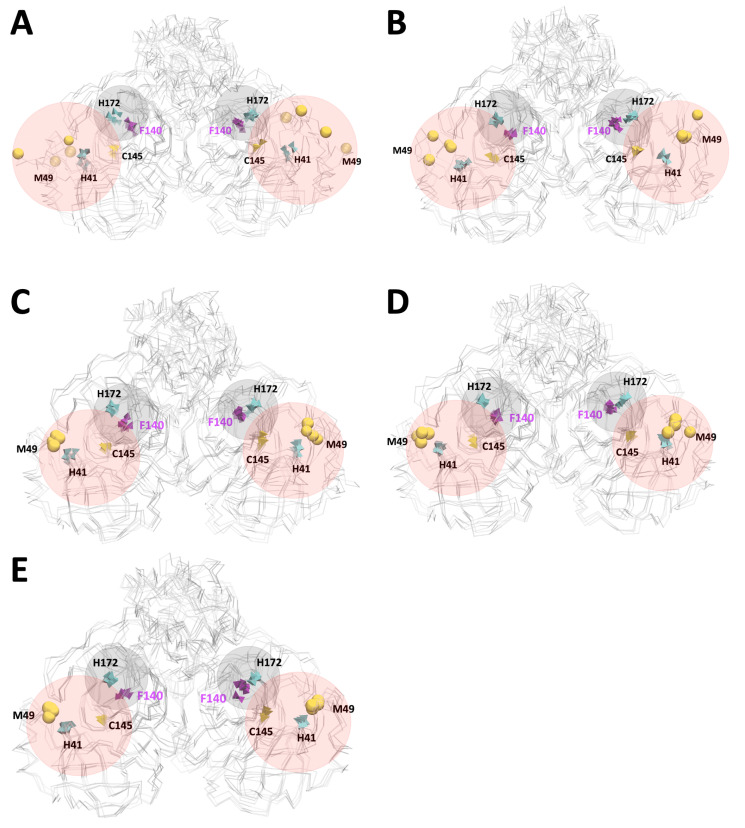
Snapshots of the last frame of all microstates in Mpro, highlighting the active site (pink area) and pocket S1 (gray area). (**A**,**B**) pH 3 and pH 4 did not show a constant Met49–Cys145 distance, which was also analysed in the RMSF, where the Met49 residue showed high fluctuations at these pHs. (**C**–**E**) pH 5, pH 6 and pH 7 with a constant distance between Met49-Cys145, His41-Cys145 and His172-Phe140. The His41, His172, Cys145 and Phe49 residues (polyhedral graphic representation) and Met49 (bead graphic representation) are shown.

The flexibility of residues close to the active site was the object of interest and for other studies dedicated to the same stated problem. The S1 pocket was crucial in the protein dimerisation and residues involved in the substrate-binding sites of SARS-CoV-2 Mpro. For these reasons, we focused on the residues of the catalytic dyad (Cys145 and His41), two residues of the S1 pocket (His172 and Phe140) proposed by Verma et al. [54] and additionally the most fluctuating residues of RMSF analysis (Met49 and Gln189). In Figure 6, the relative distance of His41 and Met49 to Cys145 was analysed for the last frame of each microstate. The most remarkable difference is observed at pH 3 and pH 4, where Met49 is located with the most significant distance to Cys145 (in both protomers). Despite this behaviour in Met49, the other residues (His41, Cys145, Phe140, and His172) were not altered by changing the pH.

The calculation of the average distance of the last 100ns between Cα atoms showed us a constant distance value between Cys145-His41 and His172-Phe140; therefore, the active centre and pocket S1 of Mpro is not significantly altered by pH effect. However, the average distance between Met49 and Gln189 shows significant differences at pH 3 (see Figure S2 in the Supporting Information for more details). This final result indicates that the pocket increases its volume as there is a greater distance between these residues at pH 3 and pH 4. Therefore, the size of the pocket can compromise the selectivity of the substrate.

Two main steps are identified in the catalytic dyad: the acylation (acyl-enzyme complex) and deacylation (acyl-enzyme hydrolysed). Świderek and Moliner analysed the deacylation step mediated by a water molecule activated by His41 [95]. Therefore we have evaluated how the pH affects the solvation of the reactive atoms of this catalytic residue.

**Figure 7:**
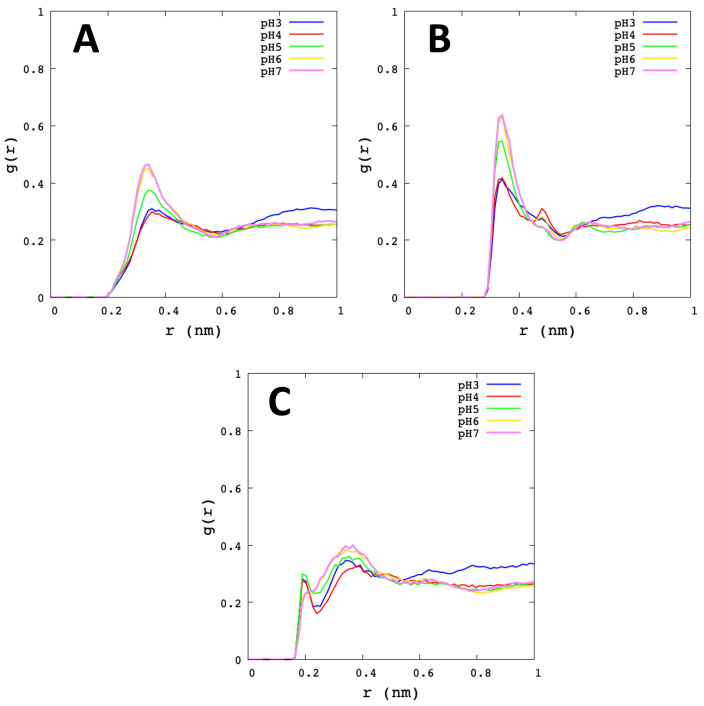
Radial distribution function of Cys145. The graphs show the average value of the Mpro protomers and the different microstates for each pH. (**A**) Sγ–Hω. (**B**) Sγ–Oω. (**C**) Hγ–Oω.

The radial distribution function (RDF) is the probability distribution to find a particle at a distance *r* away from a given reference particle. In this work, the RDF of specific atoms of Cys145 (Sγ, Hγ), His41 (Nδ1, Hδ1), Met49 (Sδ), water sites (Hω, Oω), and ions, were analysed. In Figure 7, the high of the first peak-height of Sγ–Hω and Sγ–Oω appear at *r* distance of 0.36 nm and 0.34 nm, respectively. The most striking variation is seen at pH 7 and pH 6, followed by pH 5 and lower peaks at pH 4 and pH 3. In general, the RDF of the solvent distribution on Sγ and Hγ shows a low water affinity at low pHs, which could be crucial to understand the role of environmental conditions in the catalytic mechanism of SARS-CoV-2 Mpro. Additionally, the RDF analysis of ions around Sγ from Cys145, shows the peak height at *r* distance of 0.5 nm for pH 3 and pH 4. It is worth noting that there is a significatively presence of mobile ions close to the active site (see Figure S3a from Supporting Information).

**Figure 8:**
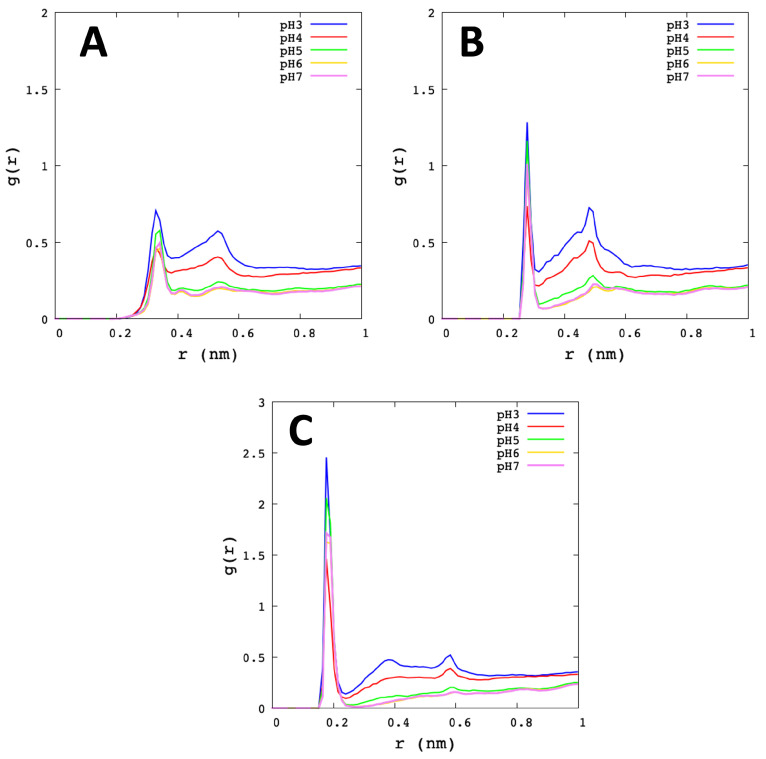
Radial distribution function of His41. The graphs show the average value of the Mpro protomers and the different microstates for each pH. (**A**) Nδ1–Hω. (**B**) Nδ1–Oω. (**C**) Hδ1–Oω.

His41 reported more affinity for water at pH 3 than pH 7 (Figure 8). Indeed, Nδ1–Hω and Nδ1–Oω show the high first of the peak-height at pH 3 with *r* = 0.33 nm and *r* = 0.28 nm, respectively. Likewise, the Hδ1–Oω showed a large peak at *r* distance of 0.18 nm, where the highest was pH 3. Therefore, with this result, we can observe that the Nδ1 and Hδ1 atoms from His41 are more related to water at low pHs. Furthermore, His41 with ions showed a similar behaviour to Cys145 (see Figure S3b from Supporting Information).

**Figure 9:**
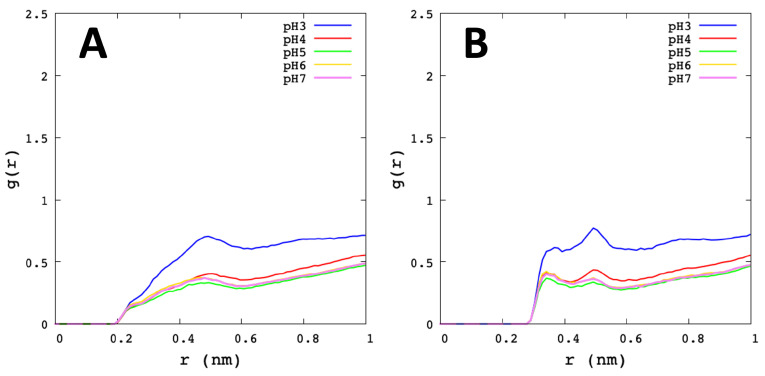
Radial distribution function analysis of Met49. The graphs show the average value of the Mpro protomers and the different microstates for each pH. (**A**) Sδ–Hω. (**B**) Sδ–Oω.

The RDF analysis of Met49 is seen in Figure 9. The Sδ–Hω obtained the peak-height at *r* distance of 0.49 nm, similar to Sδ–Oω at pH 3. Although Met49 is highly fluctuating at pH 3, this does not influence the early interaction with water molecules.

In summary, all findings of MD simulations allowed us to understand the high stability of Mpro at low conditions of pHs. The conformational stability of Mpro during the simulation time remains stable at the explored pHs scales. The significant number of residues at low pHs led to increased SASA. The impact of this effect was translated to hydrogen bond formation (intramolecular and intermolecular), which was confined. Although these results are not unexpected, they show that the ensemble of the Mpro dimer remains stable and that the most acidic pH conditions seem to be unable to disaggregate the enzyme. Furthermore, we have shown that the distance between the residues of the active site and the S1 pocket of the Mpro remained constant during the simulation time, which indicated that the structure of Mpro could not disaggregate at low pH conditions. On the contrary, the residues of the zone of binding-substrate supported conformational changes at pH 3, which increased the pocket volume. This result has related to the high fluctuations are presented in the RMSF diagram. Taking into account the importance of the solvent in the reaction mechanism of SARS-CoV-2 Mpro. In the RDF analysis, we have found that the solvent showed a high and low affinity towards the atoms of His41 and Cys145, respectively. Finally, the last frame of each trajectory was utilised to analyse the accessible values of the torsion angles ϕ and ψ to create a Ramachandran plot. This analysis allowed us to validate the protein structures of each pH. Mpro at different pHs conditions (3 to 7) had a similar percentage of residues in allowed (∼99%) and favoured (∼92%) regions (more details are in Table S5 from Supporting Information).

### 3.3. Molecular Docking Analysis

To analyse the effect of environmental pH in the interaction with protease inhibitors, a recent ligand of Mpro protease has been selected in the docking study. Molecular docking calculations with PF-00835231 ligand [19] and the 40 receptors (last frame of five pHs *x* five microstates *x* two monomers) in different conformations according to the protonation states have been conducted. (See Figure S4 from Supporting Information, where PF-00835231 presents an estimated pKa equal to 12.76 ± 0.10 predicted using ACDlabs software v11.02, which allows it to be neutral and does not suffer alteration with the change in pH.) Appropriated protonation states were preserved to both receptors and ligand. Both the number of flexible side chains and the size of the search domain were different for all the cases because of the receptor’s conformation (i.e., chain orientation, the position of residues). On average, there were about 30 flexible side chains (from about 27 to about 35 flexible side chains). Table S6 from Supporting Information represents the data for the SARS-CoV-2 Mpro with different pH: sizes of the search domain, the volume of the search domain, and the number of the flexible side chains.

The search space was defined by a docking box that wraps the space around the receiver for each system with 40 different docking boxes. Active pocket amino acid residues were used to centre the docking box. Therefore, the pocket was placed around Cys145.

**Figure 10:**
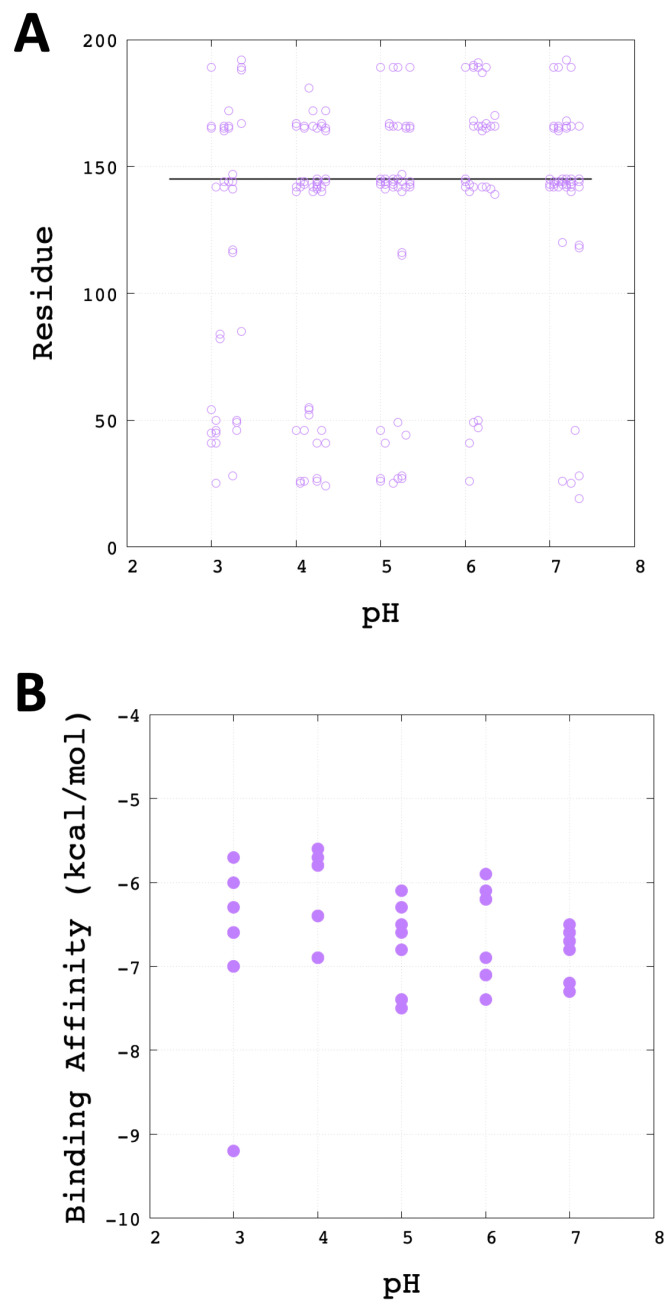
Binding analysis. (**A**) Mpro residues that interact with the drug PF-00835231 through hydrogen bonds at different pH (3, 4, 5, 6 and 7). For each pH, the best docking of each of the 8 microstates is analysed. To distinguish between the different docking poses, each is shifted slightly on the pH axis. A horizontal line is also depicted at 145 to help visualise the interaction with the catalytic Cys145. (**B**) The predicted binding affinity of drug PF-00835231 with Mpro at different pH. For each pH, the best binding affinity of the 8 different microstates is shown.

In AutoDock Vina the electrostatic interactions were mainly determined through hydrogen bonding terms. Figure 10A shows the residues of Mpro that establish hydrogen bonds with the PF-00835231 drug at different pHs (Figure S5 shows the 2D representation of the best coupling of each pH). Furthermore, at pH3, an additional pattern of interaction could be seen that shows the interaction of the drug with residues Met82, Asn84 and Cys85. On the other hand, Figure 10B shows the binding affinity of the best docking poses for each microstate. The binding mode of the drug close to the catalytic Cys145 shows a similar range of affinity (−7 to −6 kcal/mol) among all studied pHs. However, the more acidic pH shows an additional mode of interaction, being more separated from the catalytic Cys145 with the highest affinity.

**Figure 11:**
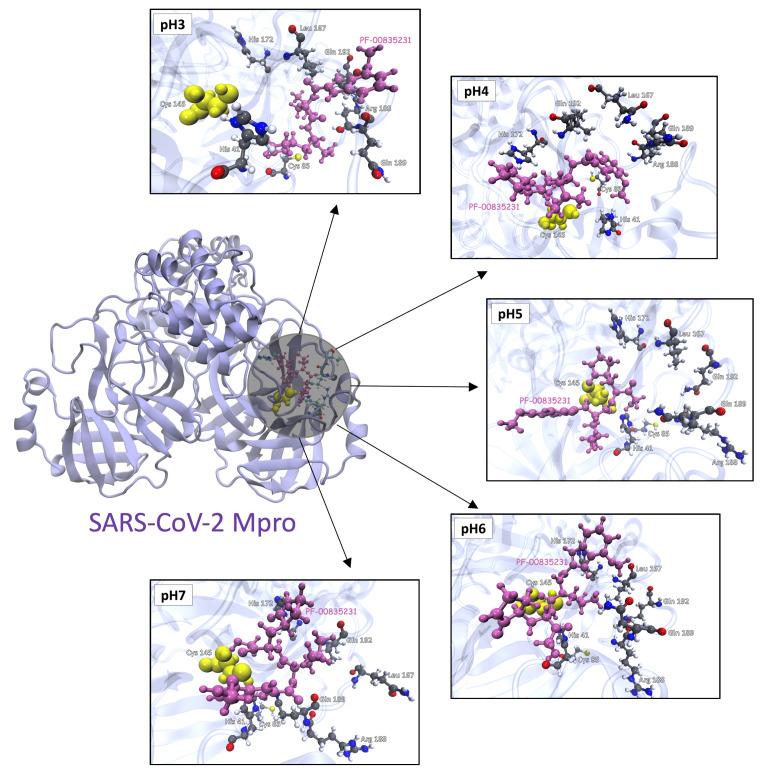
Docking snapshot of the best binding affinity at different pHs between PF-0085231 and SARS-CoV-2 Mpro. The Cys145 is highlighted in yellow and the PF-00835231 drug is highlighted in pink. The ligand is located close to the SARS-CoV-2 Mpro active site at different conditions of pHs.

According to the docking results, the Figure 11 reports the best coupling energies for each system studied. Where the His41 and Cys145 residues remain very close to PF-00835231 at different pH conditions.

## 4. Conclusions

The SARS-CoV-2 Mpro is an exciting target gaining the attention of many research groups globally. Its primary importance lies in the replication of the virus, and its inhibition could be the key to stopping the virus from spreading. However, one of the parameters in which is compromised the activity of this enzyme is the pH-dependence.

Although from the point of view of experimental analysis, the study of proteins under low pH conditions is a great challenge. The computational chemistry approach looks the way to study the effect of pH on many important mechanisms such as explored herein.

The program was developed to define a total charge distribution for a specific pH, developed under the SGCMC method. This work showed the distribution of protonation/deprotonation states of the titratable residues with a total net positive charge in pH 3 until pH 5 and a total net negative charge at pH 6 and 7. Moreover, this analysis was able to estimate the isoelectric point (pI = 5.5) computationally. The conventional all-atom MD showed us that Mpro has a stable at low pH structure.

Indeed, Mpro at pH 4 to 7 showed high structural stability of the whole protein, active site, permitting the promoters’ dimerisation. For the case of microstates at pH 3, slight conformational changes were observed. This pH-dependent computational approach revealed that Met49 showed the most significant fluctuations at pH 3 and increased the Mpro pocket volume. Identifying these mechanisms could be used for target selectivity validation.

The last part of the paper elucidates the druggability interface for protonation-dependent Mpro binding sides for a drug candidate in the clinical trial phase. The protein surface was explored using a molecular docking-based screen for a new drug candidate (PF-00835231). The predicted binding modes and affinity for the drug candidate were found in the range from pH 4 to pH 7. While at pH 3, an additional mode of binding was observed; however, more studies could be necessary to elucidate its relevance in Mpro inhibition at very acidic pH.

## Figures and Tables

**Table 1:** Total charge of Mpro as a function of pH for the different microstates (MicroS).

	MicroS-1	MicroS-2	MicroS-3	MicroS-4	MicroS-5	MicroS-6	MicroS-7	MicroS-8	MicroS-9	MicroS-10	Average Value
pH3	38	40	41	42	43	43	42	41	39	41	41 ± 2
pH4	24	27	20	22	23	20	26	22	22	22	23 ± 2
pH5	7	4	8	6	2	5	1	5	2	7	5 ± 2
pH6	−6	−5	−4	−4	−5	−6	−8	−7	−4	−6	−(5.5 ± 1.4)
pH7	−8	−9	−9	−8	−8	−7	−8	−9	−9	−9	−(8.4 ± 0.7)

**Table 2:** Values of the MD were fitted against the average structure of the last 100 ns of the dynamics was used.

pH	RMSD	RG	SASA	Hbond	Hbond
	(nm)	(nm)	(nm${}^{2}$)	(Protein–Protein)	(Protein–Waters)
pH3	0.26 ± 0.06	2.553 ± 0.010	268 ± 2	401 ± 1	(107 ± 4) × 10
pH4	0.20 ± 0.02	2.538 ± 0.002	261 ± 2	437 ± 10	(110 ± 2) × 10
pH5	0.21 ± 0.04	2.537 ± 0.005	259 ± 3	448 ± 3	(114 ± 8) × 10
pH6	0.20 ± 0.02	2.532 ± 0.005	260 ± 2	456 ± 6	(114 ± 12) × 10
pH7	0.19 ± 0.02	2.526 ± 0.007	259 ± 1	461 ± 1	(114 ± 12) × 10

## Data Availability

The access code of the Mpro SARS-CoV-2 crystal structure of this study is described in the Computational Methods Section. The homemade developed code is freely available at GitHub https://github.com/smadurga/Protein-Protonation.

## Supplementary Materials

The following are available online at https://www.mdpi.com/article/10.3390/polym13213823/s1, Figure S1: Plot of hydrogen bonds and SASA during the time of MD simulations at different pHs. (a) Solvent Accessible Surface Area. (b) Intramolecular Hydrogen bonds between protein-protein. (c) Intermolecular Hydrogen bonds between protein-waters, Figure S2: Distances between C-atoms from residues of the active site (Cys145 and His41) and S1 pocket in Mpro. (a) Average distance Cys145-His41. (b) Average distance of Met49-Gln189. (c) Average distance of His172-Phe140, Figure S3: Radial Distribution of active site residues against Mpro. (a) S*γ*–ions of Cys145. (b) N*δ*1–ions of His41. (c) S*γ*–ions of Met49, Figure S4: 2D representation of PF-00835231, Figure S5: 2D representation of the best binding affinity Mpro–PF-00835231. (a) pH3. (b) pH4. (c) pH5. (d) pH6. (e) pH7, Table S1: Total charge of each titratable amino acids, Table S2: Protonate/deprotonate microstate of His41 and His172. The symbol P and Ddefine the protonated and deprotonated states, respectively, Table S3: Values of the MD was fitted against the average structure of the last 100 ns of the dynamics was used, Table S4: Distances between amino acids from the catalytic dy-ad for each microstate, Table S5: Numerical values of Ramachandran plot of the last frame of each microstate determined by Molprobity server, Table S6: Docking results of each microstate at different pHs.

## Abbreviations

The following abbreviations are used in this manuscript:

SARS-CoV-2	Severe Acute Respiratory Syndrome Coronavirus 2
Mpro	Main Protease
SGCMC	Semi-Grand Canonical Monte Carlo
MD	Molecular Dynamics
RMSD	Root-Mean Squared Deviation
RMSF	Root-Mean Squared Fluctuation
RG	Radius of Gyration
HB	Number of Hydrogen Bonds
SASA	Solvent Accessible Surface Area
RDF	Radial Distribution Function
