# Supplementary material for: Unveiling the Effect of Low pH on the SARS-CoV-2 Main Protease by Molecular Dynamics Simulations
Source: Polymers (Basel). 2021 Nov 5;13(21):3823. doi: 10.3390/polym13213823 (PMC8587287; doi:10.3390/polym13213823)
Supplement: Supplementary file 1 [file polymers-13-03823-s001.zip › polymers-1393708-supplementary.pdf]

# Supporting Information: Unveiling the Effect of Low pH on the SARS-CoV-2 Main Protease by Molecular Dynamics Simulations

Haruna L. Barazorda-Ccahuana,[†,‡] Miroslava Nedyalkova,[¶] Francesc Mas,[*,†] and Sergio Madurga[*,†]

†*Materials Science and Physical Chemistry Department & Research Institute of Theoretical and Computational Chemistry (IQTCUB), University of Barcelona, Barcelona, Spain*

‡*Vicerrectorado de Investigación, Universidad Católica de Santa María, Arequipa, Perú*

¶*Department of Inorganic Chemistry, University of Sofia "St. Kl. Okhridski", Sofia, Bulgaria*

E-mail: fmas@ub.edu; s.madurga@ub.edu

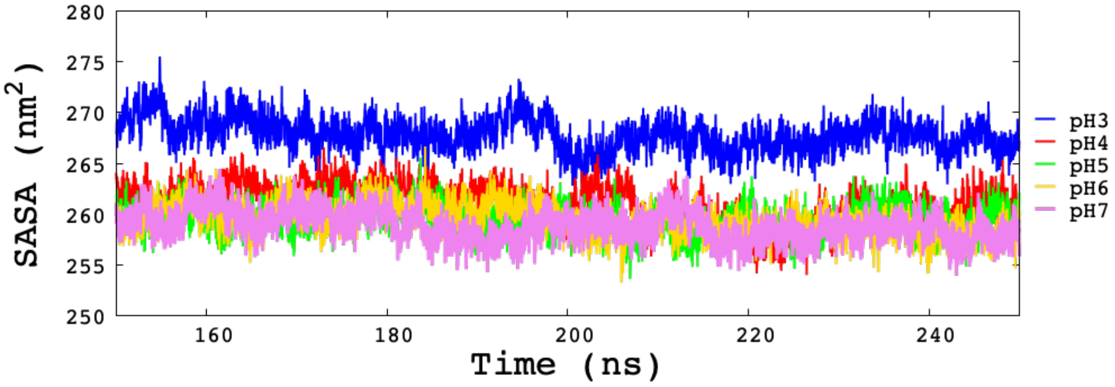


(a)

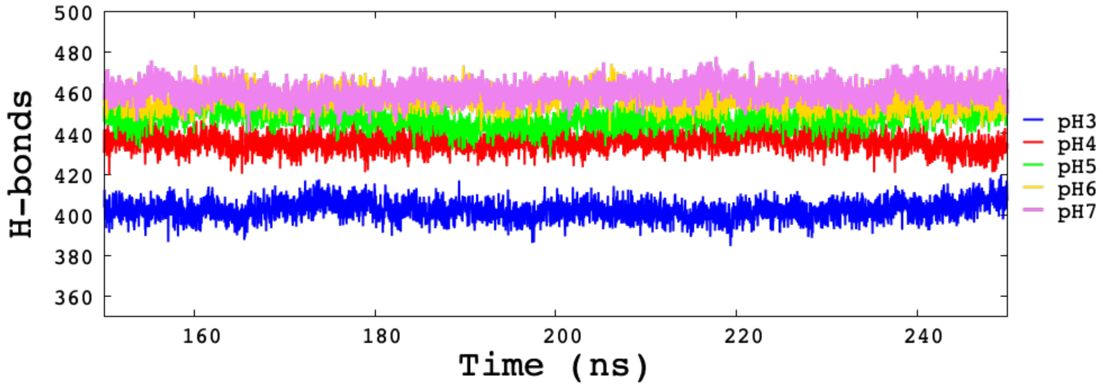


(b)

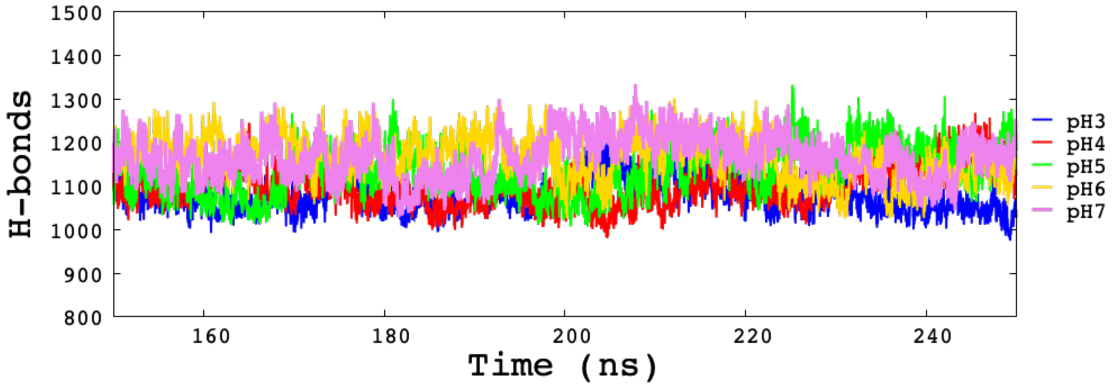


(c)

**Figure S1.** Plot of hydrogen bonds and SASA during the time of MD simulations at different pHs. (a) Solvent Accessible Surface Area. (b) Intramolecular Hydrogen bonds between protein-protein. (c) Intermolecular Hydrogen bonds between protein-waters.

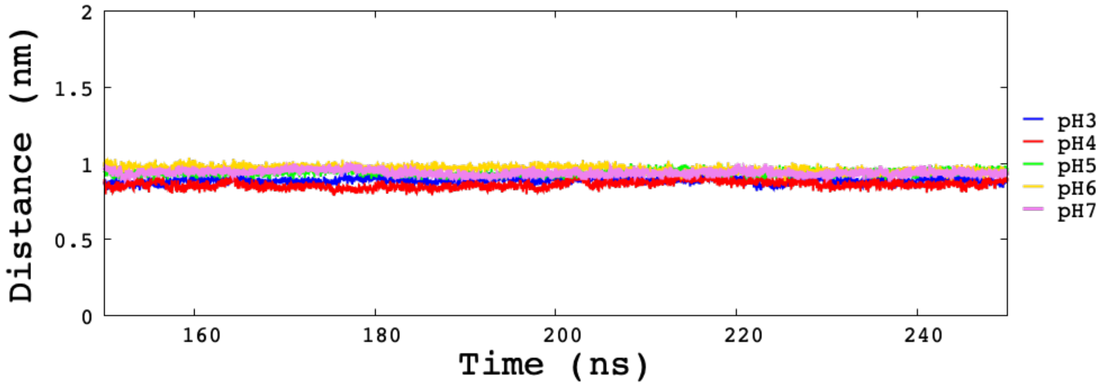


(a)

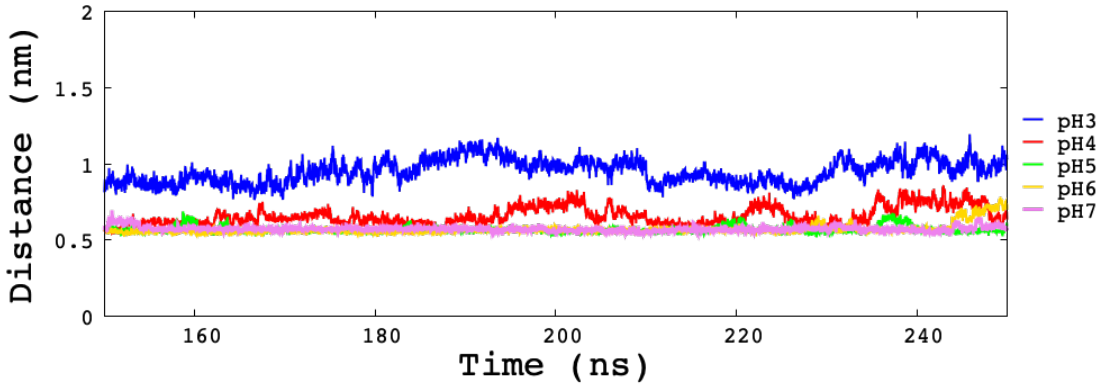


(b)

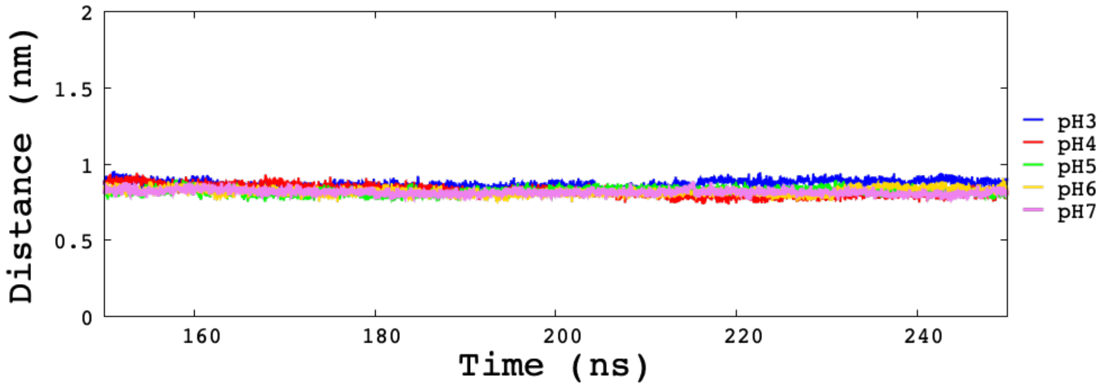


(c)

**Figure S2.** Distances between C-$\alpha$ atoms from residues of the active site (Cys145 and His41) and S1 pocket in Mpro. (a) Average distance Cys145-His41. (b) Average distance of Met49-Gln189. (c) Average distance of His172-Phe140.

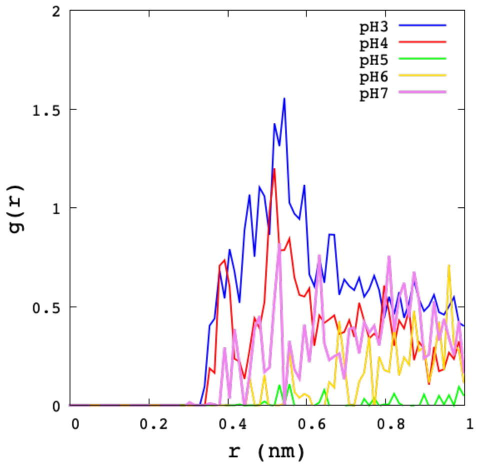


(a)

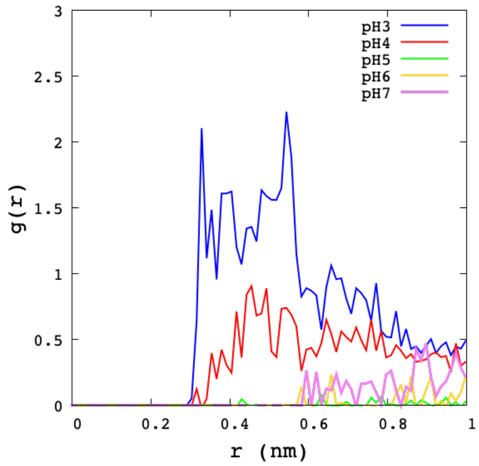


(b)

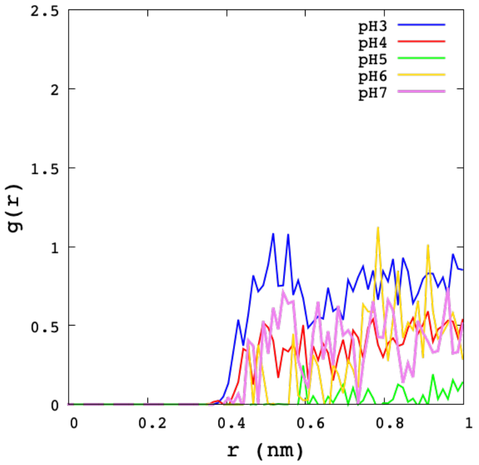


(c)

**Figure S3.** Radial Distribution of active site residues against Mpro. (a) S$\gamma$–ions of Cys145.(b) N$\delta$1–ions of His41. (c) S$\gamma$–ions of Met49.

**Table S1.** Total charge of each titratable amino acids.

| Residues | MicroS-1 | MicroS-2 | MicroS-3 | MicroS-4 | MicroS-5 | MicroS-6 | MicroS-7 | MicroS-8 | MicroS-9 | MicroS-10 | Average value |
|---|---|---|---|---|---|---|---|---|---|---|---|
| **pH3** | | | | | | | | | | | |
| ASP (34) | -11 | -9 | -9 | -8 | -8 | -8 | -9 | -7 | -10 | -8 | **-(9±1)** |
| GLU (18) | -5 | -5 | -3 | -4 | -3 | -3 | -3 | -6 | -5 | -5 | **-(4±1)** |
| ARG (22) | 22 | 22 | 22 | 22 | 22 | 22 | 22 | 22 | 22 | 22 | **22** |
| LYS (22) | 22 | 22 | 22 | 22 | 22 | 22 | 22 | 22 | 22 | 22 | **22** |
| HIS (14) | 10 | 10 | 9 | 10 | 10 | 10 | 10 | 10 | 10 | 10 | **10** |
| **pH4** | | | | | | | | | | | |
| **Residues** | **MicroS-1** | **MicroS-2** | **MicroS-3** | **MicroS-4** | **MicroS-5** | **MicroS-6** | **MicroS-7** | **MicroS-8** | **MicroS-9** | **MicroS-10** | **Average** |
| ASP (34) | -21 | -21 | -27 | -22 | -22 | -24 | -20 | -26 | -22 | -24 | **-(23±2)** |
| GLU (18) | -6 | -6 | -6 | -9 | -8 | -8 | -6 | -6 | -7 | -6 | **-(7±1)** |
| ARG (22) | 22 | 22 | 22 | 22 | 22 | 22 | 22 | 22 | 22 | 22 | **22** |
| LYS (22) | 22 | 22 | 22 | 22 | 22 | 22 | 22 | 22 | 22 | 22 | **22** |
| HIS (14) | 9 | 10 | 9 | 9 | 9 | 9 | 9 | 10 | 8 | 8 | **9±1** |
| **pH5** | | | | | | | | | | | |
| **Residues** | **MicroS-1** | **MicroS-2** | **MicroS-3** | **MicroS-4** | **MicroS-5** | **MicroS-6** | **MicroS-7** | **MicroS-8** | **MicroS-9** | **MicroS-10** | **Average** |
| ASP (34) | -32 | -30 | -34 | -33 | -31 | -33 | -30 | -34 | -33 | -32 | **-(32±1)** |
| GLU (18) | -9 | -13 | -7 | -9 | -12 | -11 | -15 | -10 | -12 | -10 | **-(11±2)** |
| ARG (22) | 22 | 22 | 22 | 22 | 22 | 22 | 22 | 22 | 22 | 22 | **22** |
| LYS (22) | 22 | 22 | 22 | 22 | 22 | 22 | 22 | 22 | 22 | 22 | **22** |
| HIS (14) | 6 | 5 | 7 | 5 | 3 | 7 | 4 | 7 | 5 | 7 | **6±1** |
| **pH6** | | | | | | | | | | | |
| **Residues** | **MicroS-1** | **MicroS-2** | **MicroS-3** | **MicroS-4** | **MicroS-5** | **MicroS-6** | **MicroS-7** | **MicroS-8** | **MicroS-9** | **MicroS-10** | **Average** |
| ASP (34) | -34 | -34 | -34 | -33 | -33 | -34 | -34 | -34 | -34 | -33 | **-(34±1)** |
| GLU (18) | -16 | -16 | -14 | -16 | -15 | -16 | -17 | -17 | -15 | -16 | **-(16±1)** |
| ARG (22) | 22 | 22 | 22 | 22 | 22 | 22 | 22 | 22 | 22 | 22 | **22** |
| LYS (22) | 22 | 22 | 22 | 22 | 22 | 22 | 22 | 22 | 22 | 22 | **22** |
| HIS (14) | 2 | 3 | 2 | 3 | 1 | 2 | 1 | 2 | 3 | 1 | **2±1** |
| **pH7** | | | | | | | | | | | |
| **Residues** | **MicroS-1** | **MicroS-2** | **MicroS-3** | **MicroS-4** | **MicroS-5** | **MicroS-6** | **MicroS-7** | **MicroS-8** | **MicroS-9** | **MicroS-10** | **Average** |
| ASP (34) | -34 | -34 | -34 | -34 | -34 | -34 | -34 | -34 | -34 | -34 | **-34** |
| GLU (18) | -17 | -17 | -17 | -16 | -17 | -17 | -16 | -17 | -18 | -18 | **-(17±1)** |
| ARG (22) | 22 | 22 | 22 | 22 | 22 | 22 | 22 | 22 | 22 | 22 | **22** |
| LYS (22) | 22 | 22 | 22 | 22 | 22 | 22 | 22 | 22 | 22 | 22 | **22** |
| HIS (14) | 1 | 0 | 0 | 0 | 1 | 2 | 0 | 0 | 1 | 1 | **1±1** |

**Table S2.** Protonate/deprotonate microstate of His41 and His172. The symbol P and D define the protonated and deprotonated states, respectively.

| **Residue** | **MicroS** | **pH3** | **pH4** | **pH5** | **pH6** | **pH7** |
|---|---|---|---|---|---|---|
| His41 | 1 | chainA(P)/chainB(P) | chainA(P)/chainB(D) | chainA(D)/chainB(D) | chainA(D)/chainB(D) | chainA(D)/chainB(D) |
| | 2 | chainA(P)/chainB(P) | chainA(P)/chainB(P) | chainA(D)/chainB(D) | chainA(D)/chainB(D) | chainA(D)/chainB(D) |
| | 3 | chainA(D)/chainB(P) | chainA(D)/chainB(P) | chainA(P)/chainB(P) | chainA(D)/chainB(D) | chainA(D)/chainB(D) |
| | 4 | chainA(P)/chainB(P) | chainA(P)/chainB(P) | chainA(D)/chainB(D) | chainA(D)/chainB(D) | chainA(D)/chainB(D) |
| His172 | 1 | chainA(P)/chainB(P) | chainA(P)/chainB(P) | chainA(P)/chainB(P) | chainA(D)/chainB(D) | chainA(D)/chainB(D) |
| | 2 | chainA(P)/chainB(P) | chainA(P)/chainB(P) | chainA(D)/chainB(D) | chainA(D)/chainB(D) | chainA(D)/chainB(D) |
| | 3 | chainA(P)/chainB(P) | chainA(P)/chainB(P) | chainA(D)/chainB(D) | chainA(D)/chainB(D) | chainA(D)/chainB(D) |
| | 4 | chainA(P)/chainB(P) | chainA(P)/chainB(P) | chainA(D)/chainB(D) | chainA(D)/chainB(D) | chainA(D)/chainB(D) |

**Table S3.** Values of the MD was fitted against the average structure of the last 100 ns of the dynamics was used.

| **pH** | **MicroS** | **RMSD (nm)** | **RG (nm)** | **SASA ($nm^2$)** | **Hbond (protein-protein)** | **Hbond (protein-waters)** |
|---|---|---|---|---|---|---|
| pH3 | pH3-1 | 0.28±0.02 | 2.543±0.009 | 269±3 | 404±9 | (111±9)x10 |
| | pH3-2 | 0.21±0.02 | 2.561±0.011 | 269±3 | 409±10 | (103±5)x10 |
| | pH3-3 | 0.32±0.05 | 2.543±0.019 | 266±5 | 393±10 | (110±8)x10 |
| | pH3-4 | 0.24±0.02 | 2.562±0.008 | 267±3 | 405±9 | (109±9)x10 |
| | Average | 0.26±0.06 | 2.553±0.010 | 268±2 | 401±1 | (107±4)x10 |
| pH4 | pH4-1 | 0.22±0.02 | 2.537±0.008 | 261±3 | 438±10 | (109±10)x10 |
| | pH4-2 | 0.21±0.02 | 2.538±0.008 | 262±3 | 427±9 | (110±10)x10 |
| | pH4-3 | 0.18±0.02 | 2.536±0.008 | 259±3 | 447±10 | (109±9)x10 |
| | pH4-4 | 0.21±0.02 | 2.541±0.009 | 263±4 | 432±10 | (112±10)x10 |
| | Average | 0.20±0.02 | 2.538±0.002 | 261±2 | 437±10 | (110±2)x10 |
| pH5 | pH5-1 | 0.25±0.03 | 2.532±0.009 | 258±3 | 448±9 | (122±15)x10 |
| | pH5-2 | 0.24±0.02 | 2.542±0.008 | 262±3 | 450±10 | (109±9)x10 |
| | pH5-3 | 0.24±0.02 | 2.518±0.008 | 256±3 | 445±10 | (107±9)x10 |
| | pH5-4 | 0.18±0.02 | 2.537±0.008 | 262±3 | 449±9 | (115±11)x10 |
| | Average | 0.21±0.04 | 2.537±0.005 | 259±3 | 448±3 | (114±8)x10 |
| pH6 | pH6-1 | 0.18±0.01 | 2.534±0.007 | 261±3 | 462±9 | (102±2)x10 |
| | pH6-2 | 0.22±0.01 | 2.527±0.007 | 258±3 | 450±9 | (122±10)x10 |
| | pH6-3 | 0.20±0.02 | 2.534±0.008 | 259±3 | 452±9 | (126±12)x10 |
| | pH6-4 | 0.21±0.02 | 2.537±0.008 | 259±3 | 458±8 | (116±11)x10 |
| | Average | 0.20±0.02 | 2.532±0.005 | 260±2 | 456±6 | (114±12)x10 |
| pH7 | pH7-1 | 0.16±0.02 | 2.533±0.010 | 259±3 | 460±10 | (102±2)x10 |
| | pH7-2 | 0.21±0.02 | 2.526±0.008 | 258±3 | 462±9 | (126±10)x10 |
| | pH7-3 | 0.21±0.03 | 2.528±0.008 | 260±3 | 460±9 | (118±12)x10 |
| | pH7-4 | 0.19±0.01 | 2.519±0.006 | 258±3 | 461±10 | (121±12)x10 |
| | Average | 0.19±0.02 | 2.526±0.007 | 259±1 | 461±1 | (114±12)x10 |

**Table S4.** Distances between amino acids from the catalytic dyad for each microstate.

| pH | MicroS | His41-Cys145 | Met49-Cys145 | His172-Cys145 |
|---|---|---|---|---|
| pH3 | pH3-1 | 0.74±0.10 | 2.50±0.09 | 0.94±0.10 |
| | pH3-2 | 0.71±0.11 | 1.59±0.18 | 0.93±0.16 |
| | pH3-3 | 0.65±0.06 | 1.39±0.04 | 0.67±0.09 |
| | pH3-4 | 0.50±0.03 | 1.37±0.05 | 0.85±0.04 |
| | Average | 0.65±0.04 | 1.71±0.05 | 0.85±0.05 |
| pH4 | pH4-1 | 0.61±0.08 | 1.62±0.14 | 1.20±0.04 |
| | pH4-2 | 0.68±0.09 | 1.31±0.08 | 1.13±0.13 |
| | pH4-3 | 0.57±0.07 | 1.53±0.19 | 1.21±0.06 |
| | pH4-4 | 0.60±0.07 | 1.39±0.05 | 1.11±0.14 |
| | Average | 0.62±0.04 | 1.46±0.07 | 1.16±0.05 |
| pH5 | pH5-1 | 0.55±0.04 | 1.41±0.06 | 0.91±0.06 |
| | pH5-2 | 0.59±0.06 | 1.41±0.06 | 0.72±0.08 |
| | pH5-3 | 0.53±0.06 | 1.43±0.07 | 0.68±0.12 |
| | pH5-4 | 0.54±0.07 | 1.43±0.06 | 0.87±0.12 |
| | Average | 0.55±0.03 | 1.42±0.03 | 0.79±0.05 |
| pH6 | pH6-1 | 0.54±0.08 | 1.43±0.06 | 1.06±0.07 |
| | pH6-2 | 0.56±0.08 | 1.41±0.06 | 0.70±0.10 |
| | pH6-3 | 0.54±0.06 | 1.41±0.07 | 0.89±0.10 |
| | pH6-4 | 0.48±0.05 | 1.49±0.05 | 1.00±0.06 |
| | Average | 0.53±0.03 | 1.43±0.03 | 0.91±0.04 |
| pH7 | pH7-1 | 0.52±0.06 | 1.40±0.06 | 0.97±0.11 |
| | pH7-2 | 0.50±0.06 | 1.46±0.07 | 0.93±0.08 |
| | pH7-3 | 0.50±0.08 | 1.39±0.07 | 0.94±0.06 |
| | pH7-4 | 0.54±0.03 | 1.38±0.06 | 0.81±0.05 |
| | Average | 0.51±0.03 | 1.41±0.03 | 0.91±0.04 |

**Table S5.** Numerical values of Ramachandran plot of the last frame of each microstate determined by Molprobity server.

| | **MicroS** | **Poor rotamers** | | **Favored rotamers** | | **Ramachandran outliers** | | **Ramachandran favored** | | **Favored** | **Allowed** |
|---|---|---|---|---|---|---|---|---|---|---|---|
| pH3 | pH3-1 | 9 | 1.80% | 452 | 90.40% | 6 | 0.99% | 563 | 93.21% | 93.20% | 99.00% |
| | pH3-2 | 13 | 2.60% | 456 | 91.20% | 5 | 0.83% | 564 | 93.38% | 93.40% | 99.20% |
| | pH3-3 | 13 | 2.60% | 456 | 91.20% | 7 | 1.16% | 565 | 93.54% | 93.50% | 98.80% |
| | pH3-4 | 10 | 2.00% | 452 | 90.40% | 6 | 0.99% | 567 | 93.87% | 93.90% | 99.00% |
| pH4 | pH4-1 | 11 | 2.20% | 455 | 91.00% | 6 | 0.99% | 565 | 93.54% | 93.50% | 99.00% |
| | pH4-2 | 10 | 2.00% | 450 | 90.00% | 5 | 0.83% | 568 | 94.04% | 94.00% | 99.20% |
| | pH4-3 | 13 | 2.60% | 445 | 89.00% | 4 | 0.66% | 558 | 92.38% | 92.40% | 99.30% |
| | pH4-4 | 10 | 2.00% | 449 | 89.80% | 4 | 0.66% | 567 | 93.87% | 93.90% | 99.30% |
| pH5 | pH5-1 | 15 | 3.00% | 450 | 90.00% | 2 | 0.33% | 552 | 91.39% | 91.40% | 99.70% |
| | pH5-2 | 14 | 2.80% | 449 | 89.80% | 2 | 0.33% | 562 | 93.05% | 93.00% | 99.70% |
| | pH5-3 | 14 | 2.80% | 454 | 90.80% | 7 | 1.16% | 553 | 91.56% | 91.60% | 98.80% |
| | pH5-4 | 11 | 2.20% | 459 | 91.80% | 2 | 0.33% | 555 | 91.89% | 91.90% | 99.70% |
| pH6 | pH6-1 | 19 | 3.80% | 439 | 87.80% | 8 | 1.32% | 556 | 92.05% | 92.10% | 98.70% |
| | pH6-2 | 13 | 2.60% | 454 | 90.80% | 6 | 0.99% | 564 | 93.38% | 93.40% | 99.00% |
| | pH6-3 | 8 | 1.60% | 451 | 90.20% | 8 | 1.32% | 554 | 91.72% | 91.70% | 98.70% |
| | pH6-4 | 12 | 2.40% | 451 | 90.20% | 5 | 0.83% | 554 | 91.72% | 91.70% | 99.20% |
| pH7 | pH7-1 | 13 | 2.60% | 448 | 89.60% | 3 | 0.50% | 554 | 91.72% | 91.70% | 99.50% |
| | pH7-2 | 17 | 3.40% | 457 | 91.40% | 0 | 0.00% | 562 | 93.05% | 93.00% | 100.00% |
| | pH7-3 | 10 | 2.00% | 450 | 90.00% | 5 | 0.83% | 554 | 91.72% | 91.70% | 99.20% |
| | pH7-4 | 7 | 1.40% | 466 | 93.20% | 5 | 0.83% | 556 | 92.05% | 92.10% | 99.20% |

**Table S6.** Docking results of each microstate at different pHs.

| Name | Size x (Å) | Size y (Å) | Size z (Å) | Search Domain Volume ($A^3$) | Number of flexible side chains |
|---|---|---|---|---|---|
| ph3-2-a | 39.2 | 31.4 | 31.7 | 39018.896 | 32 |
| ph3-2-b | 40 | 33.2 | 32 | 42496 | 32 |
| ph3-3-a | 34.3 | 31.6 | 37.7 | 40862.276 | 32 |
| ph3-3-b | 38.4 | 33.8 | 27.8 | 36082.176 | 33 |
| ph3-4-a | 30.8 | 28.7 | 35.1 | 31026.996 | 32 |
| ph3-4-b | 31.6 | 33 | 31.9 | 33265.32 | 28 |
| ph3-a | 29.4 | 44 | 33.9 | 43853.04 | 27 |
| ph3-b | 42.7 | 37.9 | 33.6 | 54375.888 | 30 |
| ph4-2-a | 29.9 | 36.4 | 30.4 | 33086.144 | 28 |
| ph4-2-b | 31 | 29.5 | 29.8 | 27252.1 | 29 |
| ph4-3-a | 27.8 | 34.5 | 30.5 | 29252.55 | 27 |
| ph4-3-b | 28.9 | 30 | 33.3 | 28871.1 | 28 |
| ph4-4-a | 27.9 | 33.9 | 27.5 | 26009.775 | 29 |
| ph4-4-b | 29.8 | 29.2 | 37.7 | 32805.032 | 29 |
| ph4-a | 40 | 38 | 43 | 65360 | 31 |
| ph4-b | 37 | 40 | 48.1 | 71188 | 32 |
| ph5-2-a | 35 | 31.6 | 32 | 35392 | 30 |
| ph5-2-b | 32.1 | 36.2 | 35 | 40670.7 | 30 |
| ph5-3-a | 30.6 | 26.1 | 32.5 | 25956.45 | 31 |
| ph5-3-b | 28 | 32.2 | 37.8 | 34080.48 | 30 |
| ph5-4-a | 35.2 | 34.8 | 27.5 | 33686.4 | 30 |
| ph5-4-b | 25.8 | 32.5 | 31.8 | 26664.3 | 31 |
| ph5-a | 37.9 | 37.1 | 47.5 | 66789.275 | 30 |
| ph5-b | 37.2 | 37.8 | 37.8 | 53152.848 | 30 |
| ph6-2-a | 28.3 | 28.5 | 34.8 | 28067.94 | 30 |
| ph6-2-b | 30.6 | 32.2 | 30.5 | 30052.26 | 29 |
| ph6-3-a | 30.8 | 34.9 | 33.2 | 35687.344 | 28 |
| ph6-3-b | 36.8 | 30.9 | 30.7 | 34909.584 | 29 |
| ph6-4-a | 30.2 | 28.5 | 43 | 37010.1 | 30 |
| ph6-4-b | 30.2 | 31.5 | 32.4 | 30822.12 | 30 |
| ph6-a | 36 | 37.6 | 36.8 | 49812.48 | 30 |
| ph6-b | 34 | 32.1 | 46.6 | 50859.24 | 31 |
| ph7-2-a | 30 | 28.3 | 33.4 | 28356.6 | 30 |
| ph7-2-b | 33.9 | 37.7 | 28.8 | 36807.264 | 29 |
| ph7-3-a | 30 | 26.4 | 37.6 | 29779.2 | 30 |
| ph7-3-b | 35.3 | 28.1 | 26 | 25790.18 | 31 |
| ph7-4-a | 25.9 | 34.8 | 30.8 | 27760.656 | 31 |
| ph7-4-b | 32.8 | 29.3 | 32.5 | 31233.8 | 31 |
| ph7-a | 37 | 34.5 | 45.7 | 58336.05 | 30 |
| ph7-b | 45.2 | 41 | 35 | 64862 | 31 |

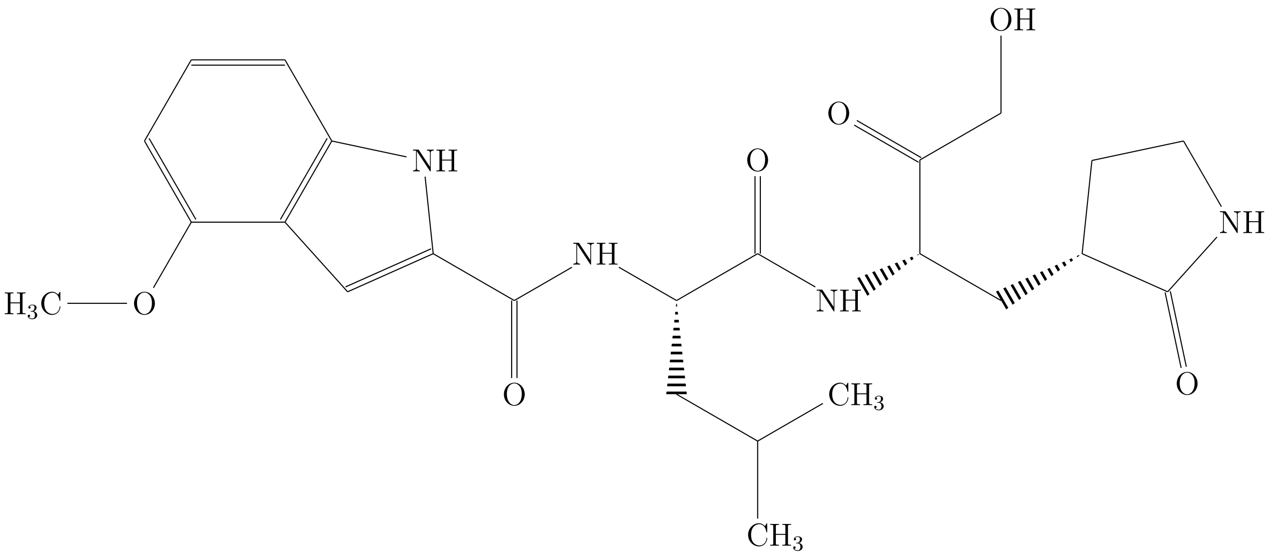


**Figure S4.** 2D representation of PF-00835231.

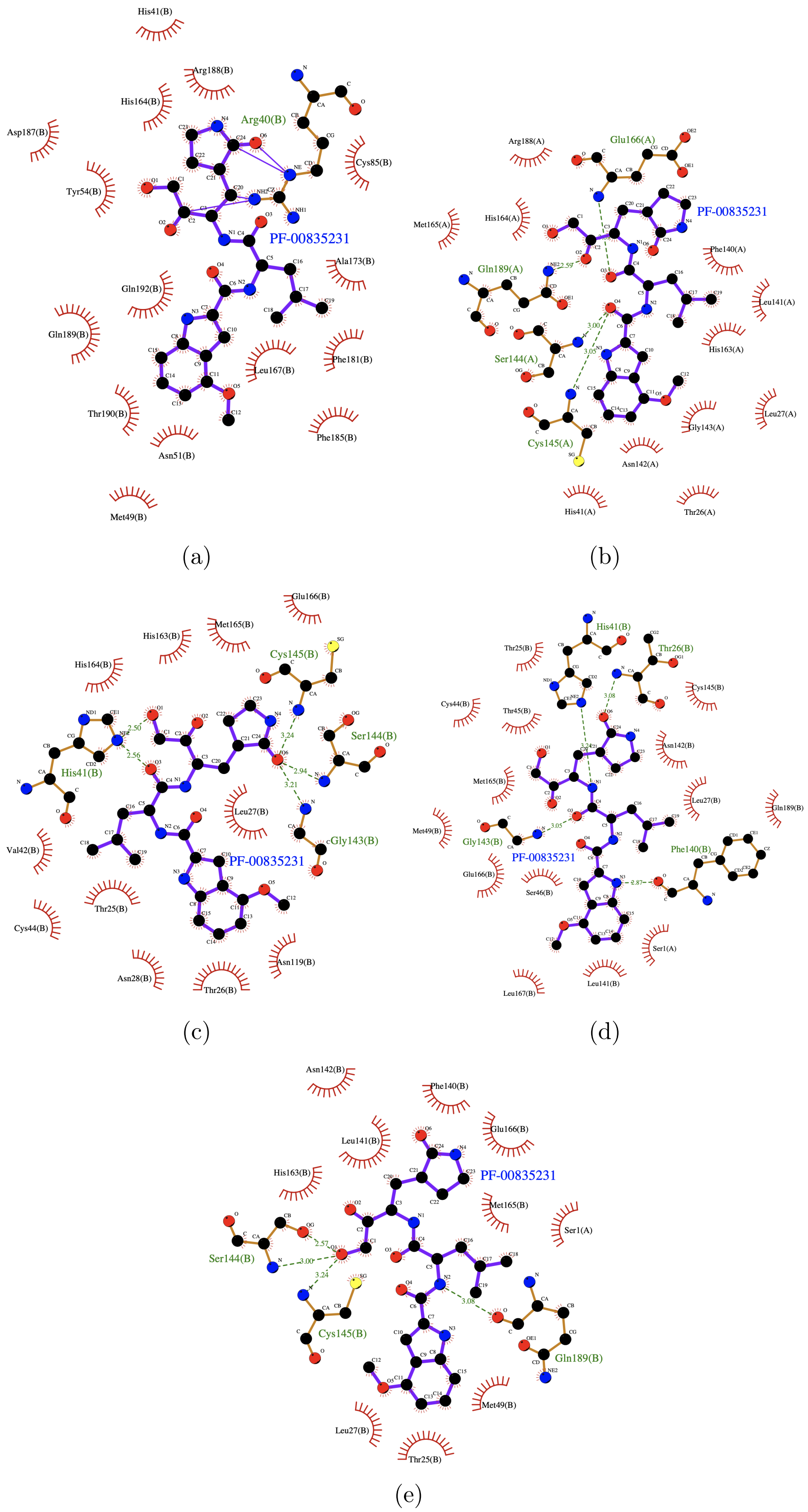


**Figure S5.** 2D representation of the best binding affinity Mpro–PF-00835231 . (a) pH3. (b) pH4. (c) pH5. (d) pH6. (e) pH7.
